# Proposal to validate *Listeria swaminathanii* sp. nov. and reassign the type strain to UTK S2-0008

**DOI:** 10.1128/spectrum.04139-25

**Published:** 2026-07-23

**Authors:** Catharine R. Carlin, Lauren K. Hudson, Harleen K. Chaggar, Claire N. Schamp, Ahmed Gaballa, Renato H. Orsi, Martin Wiedmann, Thomas G. Denes

**Affiliations:** 1 Mérieux NutriSciences, Crete, Illinois, USA; 2 IEH Laboratories and Consulting, Lake Forest Park, Washington, USA; 3 Department of Food Science, University of Tennessee 736698, Knoxville, Tennessee, USA; 4 Department of Food Science, Cornell University 5922, Ithaca, New York, USA; University of Minnesota Twin Cities, St. Paul, Minnesota, USA

**Keywords:** ANI, *Listeria*, *Listeria sensu stricto*, *Listeria sensu *lato, *Listeria swaminathanii*, novel species, type strain, taxonomy, nomenclature, species description

## Abstract

*Listeria swaminathanii* UTK S2-0008, isolated from soil collected in the Nantahala National Forest in North Carolina, USA, is the only *L. swaminathanii* strain eligible to serve as the type strain, which is needed to achieve valid status. The previously effectively published type, *L. swaminathanii* FSL L7-0020^T^, and previously described strains UTK C1-0015 and UTK C1-0024 do not conform to the International Code of Nomenclature of Prokaryotes’ rules for type strains. Additionally, the currently designated type strain (FSL L7-0020^T^ = ATCC TSD-239^T^) is an atypical representative of *L. swaminathanii* as it is the only strain lacking catalase activity. Therefore, it is proposed to reassign the type to *L. swaminathanii* (UTK S2-0008^T^ = CCUG 77280^T^ = LMG 33255^T^). Whole-genome sequence-based average nucleotide identity (ANI) showed that this strain clustered with the three previously described *L. swaminathanii* strains (FSL L7−0020 = ATCC TSD-239, UTK C1-0015, and UTK C1-0024; pairwise ANI ranged from 98.71% to 98.83%). All four strains, including the one described here, could not be classified as any validly published *Listeria* species and showed the highest similarity to *Listeria marthii* (maximum ANI of 93.92%, *in silico* DNA-DNA hybridization of 56.2%). *L. swaminathanii* exhibits the phenotypic characteristics that are currently expected of the *Listeria sensu stricto* species. This species lacks phenotypic characteristics associated with *Listeria* pathogenicity (non-hemolytic and negative for phosphatidylinositol-specific phospholipase C activity); the genomes lack genes associated with virulence (all genes found on the *Listeria* pathogenicity island 1 [LIPI-1], as well as the internalin genes *inlA* and *inlB*), which support *L. swaminathanii* is nonpathogenic.

**IMPORTANCE:**

The genus *Listeria* includes species of significant relevance to food safety, environmental microbiology, and public health. Accurate species identification is critical because misidentification of nonpathogenic species as pathogenic ones can lead to unnecessary recalls and regulatory complications. The validation of *Listeria swaminathanii* sp. nov. will ensure that this species is formally recognized and has a type strain (UTK S2-0008^T^) that is representative of the species. This work strengthens diagnostic accuracy by enabling the inclusion of this species in reference databases and inclusivity studies, reducing the risk of false identification. Furthermore, the identification and characterization of *Listeria swaminathanii sp. nov.* expands our understanding of the genetic and ecological diversity within the genus *Listeria*, particularly among soil-dwelling strains.

## INTRODUCTION

*Listeria* belongs to the family *Listeriaceae*, which includes one other genus, *Brochothrix* (1–3). Prior to 2010, the *Listeria* genus was defined by six species, which have been isolated from a variety of environments throughout the world including soil, water, vegetation, silage, animal feed, and sewage (4–6). Additionally, *Listeria* are frequently isolated from food and food processing environments where they play a key role in food safety (7–9). *Listeria monocytogenes*, the type species (10, 11), is the causative agent of foodborne listeriosis, a major public health concern worldwide (12). *Listeria ivanovii* (13, 14) is also pathogenic (15); however, this species is primarily associated with disease in ruminants (16). *L. monocytogenes* is known to cohabitate with four of the species described before 2010 that show high similarity to *L. monocytogenes* (*Listeria innocua*, *L. ivanovii*, *Listeria seeligeri*, and *Listeria welshimeri*); their presence is recognized as an indicator of a potential for *L. monocytogenes* contamination (7–9). Key phenotypic characteristics that have differentiated *Listeria* from other bacteria include (i) the ability to grow at refrigeration temperatures (4°C) and (ii) motility at 25°C (1). The pathogenic species (*L. monocytogenes* and *L. ivanovii*) are phenotypically differentiated from the non-pathogens by β-hemolysis and phosphatidylinositol-specific phospholipase C (PI-PLC) enzyme activity.

Since 2010, 22 additional species have been added to the *Listeria* genus (11), resulting in substantial modifications to the characteristics that historically defined this genus. The subdivision of *Listeria* into two clades, *sensu stricto* and *sensu lato*, based on the relatedness to *L. monocytogenes*, is widely recognized (5, 17, 18). The *Listeria sensu stricto* clade consists of *L. monocytogenes* and the eight species that show high similarity to *L. monocytogenes* (i.e., the species likely to indicate an increased risk of a food safety hazard). The characteristics historically used to define the *Listeria* genus continue to define the *Listeria sensu stricto* clade (*n* = 9 species; [19–23]). Specifically, the *Listeria sensu stricto* are defined by (i) growth at 4°C, (ii) motility at 25°C (except for *Listeria immobilis* [22, 23]), (iii) inability to reduce nitrate, (iv) inability to utilize mannitol, (v) Voges-Proskauer-positive, and (vi) catalase activity (1, 5, 17). Conversely, the *Listeria sensu lato* clade, which currently consists of 19 species, is considerably divergent from *L. monocytogenes* and many lack the characteristics that once defined *Listeria* (5, 17). Notably, (i) about half (*n* = 8) do not grow at 4°C (24–31), (ii) all but *Listeria grayi* lack motility at 25°C (22–24, 32–35), (iii) a few do not reduce nitrate (*n* = 3) (24, 27, 31), and (iv) three are negative for catalase activity (30, 31, 36). Overall, the ecology of the more recently described species (natural and food processing environments [5]) is similar to the original six. While more data are needed, the distribution of many of the more recently described *Listeria* may be limited to certain environments and geographical locations (e.g., *Listeria cossartiae*—soil, North America) (5).

*Listeria swaminathanii* groups into the *Listeria sensu stricto* clade and was first effectively published in 2022 (37, 38); these publications cannot be submitted for validation because the strains are linked to a restrictive material transfer agreement (MTA). The previously described strains (FSL L7-0020^T^ [37], UTK C1-0015, and UTK C1-0024) were isolated from soil collected in a U.S. National Park, and the MTA required by the U.S. National Park Service violates the accessibility requirements specified in the International Code of Nomenclature of Prokaryotes (INCP; see “Minute 8. IJSEM matters” in the ICSP-Executive Board Meeting minutes from 29 October 2020 [39]). Therefore, since the originally designated type, FSL L7-0020^T^ (37), cannot serve as the type strain for validation per Rule 30(4) of the INCP (40), a new type strain is proposed (UTK S2-008^T^). Additionally, the original type strain, FSL L7-0020, is an atypical representative of *L. swaminathanii* as it lacks catalase activity, a phenotype currently shared by all *Listeria sensu stricto* (*n* = 10). To date, all *L. swaminathanii* strains were isolated from soil collected in the Southeast United States (Great Smoky Mountains National Park, Nantahala National Forest). Here we describe UTK S2-0008^T^ following conventional type strain methodologies and compare it to the previously described and effectively published strains.

## RESULTS AND DISCUSSION

### Genome characteristics and similarity

The complete genome of *L. swaminathanii* UTK S2-0008^T^ was generated to establish a genomic basis for species delineation and for further genomic characterization. A hybrid assembly approach was leveraged, using short Illumina reads with long Oxford Nanopore reads, resulting in a single, closed genome assembly. The genome had a length of 2.85 Mb and a G + C content of 38.5% (Table S1). This is consistent with the ranges previously reported for the *Listeria sensu stricto* species (2.8–3.1 Mb and 34.6%–41.6%, respectively [17, 22, 23]). Additionally, it contained 2,869 genes, including 2,780 CDS and 89 RNA genes (67 tRNAs, six 5S rRNAs, six 16S rRNAs, and six 23S rRNAs).

**Fig 1:**
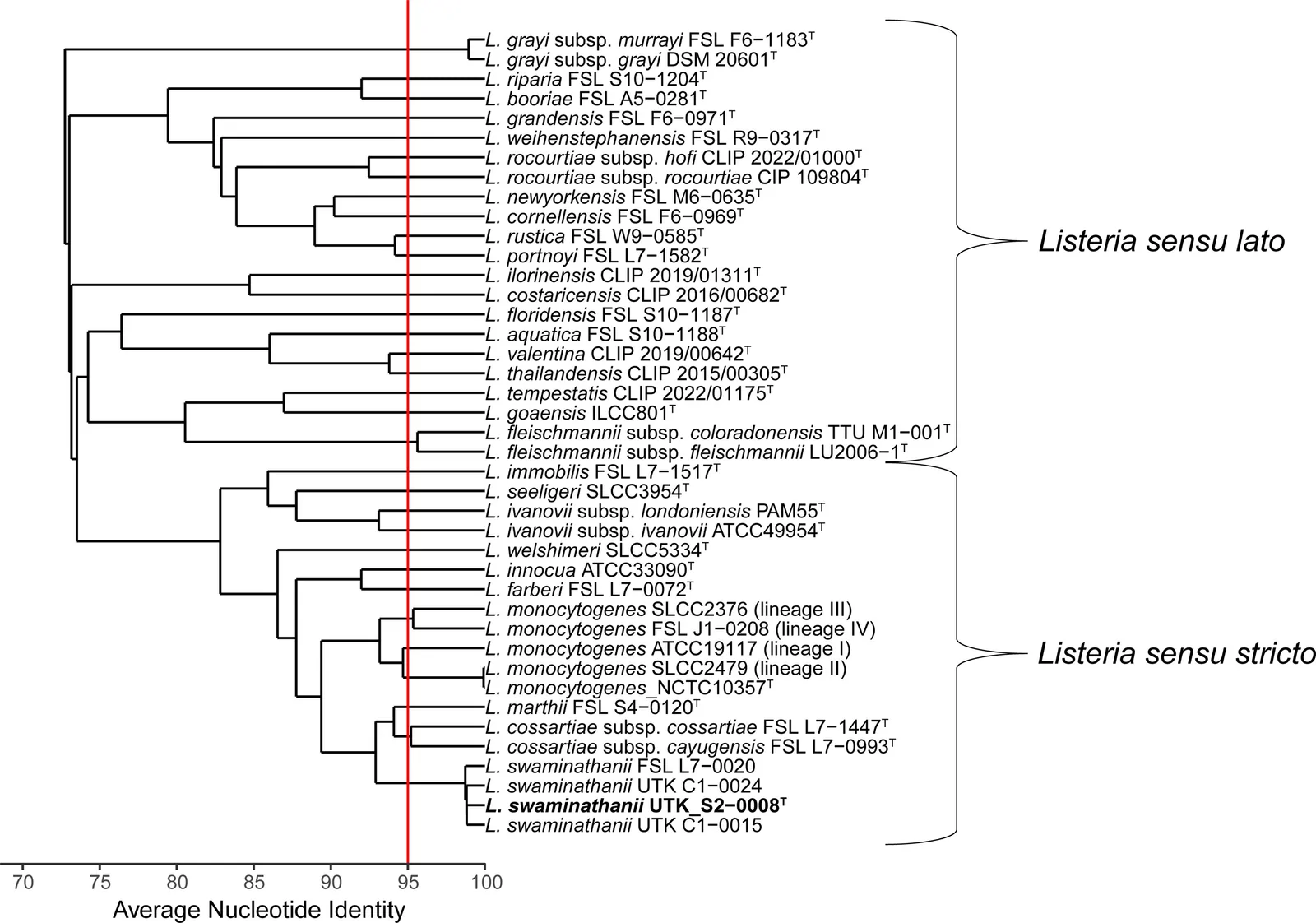
UPGMA hierarchical cluster dendrogram based on the average nucleotide identity BLAST (ANIb) analysis of two draft and two closed genomes representing the *L. swaminathanii* and 39 reference strains representing 28 *Listeria* spp. The vertical bar is placed at 95, corresponding to the proposed 95% ANIb cut-off for species differentiation (44). The type strains are identified by a superscript T. The *L. swaminathanii* type strain is bolded.

Two overall genomic relatedness indices (ORGIs) were used to assess relatedness and species delineation: Average nucleotide identity BLAST (ANIb) and *in silico* DNA-DNA hybridization (isDDH). Briefly, ANIb is the current gold standard, and isDDH is the computational version of the traditional laboratory method for assessing species placement at the genome level (41). The ANIb analysis was conducted with *L. swaminathanii* UTK S2-0008^T^, three previously characterized *L. swaminathanii* strains (UTK C1-0015, UTK C1-0024, and FSL L7-0020), and a set of 37 *Listeria* reference genomes (Fig. 1 and Table S2). The four *L. swaminathanii* strains grouped together (ANI range of 98.71%–98.83%). Furthermore, they showed the highest similarity with *Listeria marthii* (ANI range of 93.91%–93.94%; Table S2); this is below the 95%–96% threshold for species delineation (42, 43). Additionally, the isDDH values for *L. swaminathanii* UTK S2-0008^T^, FSL L7-0020, UTK C1-0015, and UTK C1-0024 compared to *L. marthii* FSL S4-120^T^ were 55.7%, 56.0%, 56.0%, and 56.2%, respectively; this is below the 70% threshold for species delineation (42, 43). These results support *L. swaminathanii* as distinct from all currently recognized *Listeria* species.

In addition, all publicly available *L. swaminathanii* genomes currently represented in the Genome Taxonomy Database (GTDB; release 11-RS232) (45) were examined; this database includes the original type strain (FSL L7-0020), UTK S2-0008^T^, and 14 other previously described *L. swaminathanii* genomes. All *L. swaminathanii* strains are assigned to a single, distinct GTDB species cluster (s__Listeria swaminathanii) that includes the original type strain is clearly separated from other closely related *Listeria* species. This GTDB species assignment, which uses ANI and alignment fraction to determine species clusters, is consistent with the ANI- and isDDH-based species delineation presented in the current study.

### Phenotypic features and species-level identification

Phenotypic features of *L. swaminathanii* UTK S2-0008^T^ were determined, including characterization tests specified in standardized *Listeria* identification procedures. *L. swaminathanii* UTK S2-0008^T^ exhibited traits consistent with its classification within the genus *Listeria* (1), including being Gram-stain-positive, short rod cell morphology, anaerobic growth at 30°C, and being oxidase negative. Additionally, most phenotypic results described here for UTK S2-0008^T^ (Table 1) are consistent with those previously reported for other *L. swaminathanii* strains (FSL L7-0020, UTK C1-0015, and UTK C1-0024) (37, 38). Shared characteristics among all strains include motility at 25°C, lack of motility at 37°C, and inability to reduce nitrate. The only observed difference was catalase activity: UTK S2-0008^T^ and the two other UTK strains are catalase positive, while FSL L7-0020 is catalase negative, a phenotype considered atypical for the species. See Table 1 for a summary of the phenotypic characterization results. All control cultures yielded the expected results.

**TABLE 1:** Phenotypic characteristics of *Listeria* species based on observations from this study and previous studies^*a*^^*b*^

Characteristics	*Sensu stricto*										*Sensu lato*																		
	Lsw sp. nov.	Lmo	Lma	Lcs	Lin	Lfr	Lws	Liv	Lse	Lim	Lgy	Lro	Lwp	Lcn	Lgd	Lri	Lbo	Lny	Lpo	Lru	Lfc	Laq	Lfl	Lgo	Lte	Lth	Lva	Lco	Lil
Motility at 25°C	+	+	+	+	+	+	+	+	+	−	+	−	−	−	−	−	−	−	−	−	−	−	−	−	−	−	−	-^†^	-^†^
Growth at 4°C	+	+	+	+	+	+	+	+	+	+	+	+	+	+	+	+	+	+	+	+	−	−	−	−	−	−	−	−	−
Nitrate reduction	−	−	−	−	−	−	−	−	−	−	V*	+	+	+	+	+	+	+	+	+	+	+	−	−	+	+	+	+	−
Voges-Proskauer	+	+	+	+	+	+	+	+	+	+	+	−	−	−	−	−	−	−	−	−	−	V	−	−	−	+	−	+	+
*β* hemolysis	−	+	−	−		−	−	+	(+)	−	−	−	−	−	−	−	−	−	−	−	−	−	−	−	−	−	−	−	−
PI-PLC	−	+	−	−	−	−	−	+	−	−	−	−	−	−	−	−	−	−	−	−	−	−	−	−	−	−	−	−	−
D-arylamidase	−	−	−	−	+	+	V	V	+	+	+	−	−	−	−	−	−	−	−	−	−	−	−	−	−	−	−	−	−
*ɑ* -Mannosidase	+	+	+	+	+	+	+	−	−	−	V	+	−	−	−	+	+	−	−	−	−	+	−	−	−	−	−	−	−
D-arabitol	+	+	+	+	+	+	+	+	+	+	+	−	+	−	V	−	+	−	(+)	(+)	+	−	−	+	+^*^	+^*^	+^*^	+	+
D-xylose	−	−	−	−	−	−	+	+	+	+	−	+	+	+	+	+	+	+	+	+	+	+	+	+	+	+	+	+	+
L-rhamnose	−	+	−	−	V	−	V	−	−	−	−	+	+	−	−	+	+	V	+	+	+	+	+	+	+	+	+	+	−
Methyl-α-D-glucopyranoside	+	+	+	+	+	+	+	+	+	+	V*	+	+	+	+	+	+	+	+	+	+	−	+	+	+	+	−	+	+
Methyl-α-D-mannopyranoside	+	+	+	+	+	+	V	−	−	−	+	−	−	−	−	−	−	−	−	−	V	−	−	−	−	−	−	+	−
D-ribose	−	−	−	V^¥^	−	−	−	V*	−	+	+	+	−	+	+	V	V	+	−	−	+	+	−	−	+	+	+	+	+
Glucose-1-phosphate	−	−	−	−	−	−	−	V	−	−	−	−	−	−	−	−	−	−	−	−	−	−	−	−	−	−	−	−	−
D-tagatose	−	−	−	−	−	−	+	−	−	−	−	−	−	−	−	−	−	−	−	−	−	+	−	−	−	+	V	−	−
Glycerol	V	V	V!	+	+	−	+	+	+	V	V	+	+	V	−	V	+	+	−	−	+	V	−	(+)	−	(+)	+	+	−
L-arabinose	−	−	−	−	−	−	−	−	−	−	−	−	−	V	−	+	+	V	+	+	−	+	+	−	−	−	+	−	−
D-galactose	−	V	−	−	−	−	−	V	−	−	+	+	−	−	−	+	+	+	+	+	−	−	+	−	−	−	−	+	+
D-glucose	+	V!	V!	+	V!	+	+	V!	+	+	+	+	+	+	+	+	+	+	+	+	+	+	+	+	+	+	+	+	+
L-sorbose	−	V!	V!	−	V!	−	−	V!	−	−	V!	−	−	−	−	−	−	−	−	−	V!	−	−	−	−	−	−	−	−
Inositol	−	−	−	−	−	−	−	−	−	−	−	−	−	−	−	V	−	−	−	V	−	V	−	−	−	+	+	−	−
D-mannitol	−	−	−	−	−	−	−	−	−	−	+	+	+	+	−	V	+	+	+	+	V	−	−	−	−	−	−	−	−
D-maltose	+	+	+	+	+	+	+	+	+	V	+	+	+	+	+	+	+	+	−	+	+	−	+	+	+	−	−	+	+
D-lactose	V	+	+	+	+	+	+	+	+	+	+	+	V!	(+)	−	+	+	+	(+)	+	−	+	+	+	−	−	−	+	+
D-melibiose	−	V!	V!	−	V	−	−	−	−	−	−	+	−	−	−	V	+	−	−	−	V	−	−	−	−	−	−	−	−
D-sucrose	−	V	−	−	+	−	+	+	+	V	−	−	−	−	−	−	−	−	−	V	−	−	−	−	+	−	−	+	−
Inulin	−	V!	−	−	V!	−	−	−	−	−	−	−	−	−	−	−	−	−	−	−	−	−	−	−	−	−	−	−	−
D-melezitose	−	V	−	−	V	−	V	V	V	V	−	−	−	−	−	−	−	−	−	−	V	−	−	−	−	−	−	−	−
D-turanose	−	−	V!	−	V	−	−	−	−	−	−	−	−	−	−	−	−	−	−	−	V*	−	−	−	−	−	−	−	−
D-lyxose	−	V	−	−	V	−	V	−	−	−	V	−	−	−	−	−	−	−	−	−	−	−	+	−	−	−	+	−	−

^*a*^ Taxa: Lsw, *L. swaminathanii *sp. nov. is highlighted in gray (this study, 37, 38); Lmo, *L. monocytogenes *(5, 10, 37, 38, 10403S, ATCC 43257); Lma, *L. marthii *(21, 24, 25, FSL S4-120^T^); Lcs, *L. cossartiae *(22, 23, FSL L7-1447^T^, FSL L7-0993^T^); Lin, *L. innocua *(19); Lfr, *L. farberi* (22, 23); Lws, *L. welshimeri *(20); Liv, *L. ivanovii *(13, 14); Lse, *L. seeligeri *(20); Lim, *L. immobilis *(22, 23); Lgy, *L. grayi *(32); Lro, *L. rocourtiae* (33, 36); Lwp, *L. weihenstephanensis *(34); Lcn, *L. cornellensis *(24); Lgd, *L. grandensis *(24); Lri, *L. riparia* (24); Lbo, *L. booriae *(35); Lny, *L. newyorkensis *(35); Lpo, *L. portnoyi *(22, 23); Lru, *L. rustica *(22, 23); Lfc, *L. fleischmannii *(25, 26); Laq, *L. aquatica *(24); Lfl, *L. floridensis *(24); Lgo, *L. goaensis *(27); Lte, *L. *tempestatis (36); Lth, *L. thailandensis *(28); Lva, *L. valentina *(29); Lco, *L. costaricensis *(30); Lil, *L. ilorinensis *(31).

^*b*^ +, positive; (+), weak positive; +^*^, positive result observed with *Listeria *API, but not API 50CH; −, negative; -^†^, *L. costaricensis *and *L. ilorinensis *are motile at 37°C, but not at 25°C; V, variable between strains; V!, variable between studies; V*, characteristics that differentiate subspecies*—L. ivanovii *subsp. *Ivanovii *ferments ribose; subsp. *londoniensis *does not ferment ribose. *L. grayi *subsp. does not reduce nitrate and ferments methyl-α-D-glucopyranoside; subsp. murrayi reduces nitrate and does not ferment methyl-α-D-glucopyranoside. *L. fleischmannii *subsp. *fleischmannii *ferments turanose; subsp. *coloradonensis. *

Growth was assessed across the range of temperatures expected for *Listeria* spp. (4°C–41°C) (1) to determine the growth temperature range and the optimal growth temperature. *L. swaminathanii* UTK S2-0008^T^ grew across the full range tested (Table 1 and Table S3), including under refrigeration temperatures, a trait of the *Listeria sensu stricto* species. Optimal growth occurred between 30°C and 37°C. Growth levels at 4°C, 22°C, 30°C, and 37°C were similar to those previously reported for *L. swaminathanii* FSL L7-0020, UTK C1-0015, and UTK C1-0024 (37, 38) (Table S3). However, growth at 41°C was strain dependent, as UTK C1-0024 did not grow at this temperature (Table S3). Overall, these results confirm that *L. swaminathanii* shares growth characteristics with other *sensu stricto* species, including the ability to grow at 4°C.

Colony morphology was assessed on selective and differential media and blood agar to confirm both the genus-level characteristics and the absence of pathogenicity indicators. Phenotypically, pathogenic species of *Listeria* are differentiated by β-hemolysis on blood agar and the hydrolysis of phosphatidylinositol on a *Listeria*-specific chromogenic medium. Modified Oxford Agar (MOX) is a selective and differential agar traditionally used to isolate *Listeria,* but it does not differentiate pathogenic species. *L. swaminathanii* UTK S2-0008^T^ generated round, brown-black colonies with a sunken center surrounded by a black zone on MOX, which is consistent with the morphology generated by all currently described *Listeria sensu stricto* species. Agar *Listeria* according to Ottaviani and Agosti (ALOA) is a chromogenic medium that detects β-glucosidase and PI-PLC enzyme activities, which are indicated by the formation of a blue-green colony surrounded by an opaque halo, respectively (46, 47). All *Listeria* described to date are positive for β-glucosidase activity. PI-PLC activity, a virulence factor, is currently only associated with the pathogenic species *L. monocytogenes* and *L. ivanovii*. When grown on ALOA, *Listeria* spp. will generate a blue-green colony; only pathogenic species will have an opaque halo indicative of PI-PLC activity. On ALOA, *L. swaminathanii* UTK S2-0008^T^ generated round, blue-green colonies without halos, which is consistent with the non-pathogenic (PI-PLC-negative, Table 1) *Listeria* spp. Sheep’s blood agar (SBA) was used to assess hemolysin activity; pathogenic species of *Listeria* will exhibit β-hemolysis, which presents as a clear zone due to lysis of the red blood cells. On SBA, *L. swaminathanii* UTK S2-008^T^ colonies were negative for β-hemolysis. The colony phenotypic results for *L. swaminathanii* UTK S2-008^T^ reported here are consistent with the results previously reported for *L. swaminathanii* FSL L7-0020, UTK C1-0015, and UTK C1-0024 (37, 38) and are typical for non-pathogenic *Listeria* spp.

*L. swaminathanii* UTK S2-0008^T^ is catalase positive. Of the four described *L. swaminathanii* strains, only FSL L7-0020 (the originally designated type strain) is catalase negative (37, 38). Specifically, further analysis of the *L. swaminathanii* genomes identified two nonsynonymous changes at codons 72 and 92 in the FSL L7-0020 *kat* sequence (37, 38). Based on current data, the absence of catalase activity is unique to FSL L7-0020 and not expected for *L. swaminathanii*. This nonconformity makes FSL L7-0020 an atypical representative of this species, while the new type strain proposed here (UTK S2-0008^T^) is phenotypically concordant with the majority of *L. swaminathanii* strains.

The API 50 CH was used to evaluate the metabolism of 50 carbohydrates and carbohydrate derivatives. Based on the API 50 CH data previously reported for UTK C1-0015, UTK C1-0024 (38), and FSL L7-0020 (37), *L. swaminathanii* strains vary in their ability to utilize glycerol, lactose, trehalose, and starch. Based on the API 50 CH results from this study and the previously reported results for *L. swaminathanii* UTK C1-0015 and UTK C1-0024 (38), *L. cossartiae* (22, 23), and *L. marthii* (24, 25), there is no phenotypic test within the API 50 CH gallery of tests that differentiate these species. A summary of the phenotypic results from the API 50 CH tests can be found in Table 1; all tests not reported in Table 1 can be found in Table S5.

The *Listeria* API kit was used for species-level identification based on enzymatic and carbohydrate fermentation tests. *L. swaminathanii* UTK S2-0008^T^ yielded the same API numerical profile (6110) previously reported for *L. marthii* (21), *L. cossartiae* (22, 23), and the three other previously described *L. swaminathanii* strains (37, 38). Since *L. marthii*, *L. cossartiae*, and *L. swaminathanii* are not represented in the API database, these species are identified as *L. monocytogenes*, primarily due to the negative DIM (differentiation *L. innocua* and *L. monocytogenes*) result. The DIM tests for D-arylamidase activity; the absence of this enzyme activity is historically used to differentiate *L. monocytogenes* from *L. innocua* (46). The API test value (*T*) for 6110 is <1 (*T* = 0.62) due to the negative result for rhamnose fermentation, which is a rare phenotype for *L. monocytogenes* and primarily associated with lineage III strains (46, 48). Importantly, this work highlights that DIM-negative strains assigned a *L. monocytogenes* API identification with numeric code 6110, but lacking the phenotypic characteristics associated with virulence (non-hemolytic, PI-PLC negative), are likely not *L. monocytogenes*.

Three rapid automated identification systems were used for species-level identification of UTK S2-0008^T^ and FSL L7-0020: VITEK 2, MITEK MS, and MALDI Biotyper. Both strains were identified as *L. innocua* by VITEK 2 (99% probability) and as *L. marthii* by VITEK MS (99% confidence; Table S4). *L. swaminathanii* FSL L7-0020 generated the same identification results by VITEK 2 and VITEK MS as previously reported (37). The MALDI Biotyper, using the full extraction procedure, identified UTK S2-0008^T^ as *L. innocua* with a high confidence score (47, 48) with the first biological replicate and with a low confidence score with the second biological replicate (Table S4). FSL L7-0020 was previously tested by MALDI Biotyper (not published) and was identified as *L. innocua* with a high confidence score (2.15); two additional biological replicates were performed for this study and yielded *L. monocytogenes* with a high confidence score and “no organism identification possible” due to a low confidence score (Table S4). These incorrect identification results are consistent with a previous study that reported misidentification of recently described *sensu stricto* species by rapid identification methods (37). As explained by Carlin et al., this issue indicates that reference databases for rapid identification methods need to be updated to include all described *Listeria* spp., particularly *sensu stricto* species, as it would be problematic for these systems to incorrectly identify nonpathogenic *Listeria* spp. as a pathogenic species like *L. monocytogenes* (37).

The *L. swaminathanii* genomes (UTK S2-0008^T^, FSL L7-0020, UTK C1-0015, and UTK C1-0024) were analyzed using the PCR serogroup scheme that is based on the Doumith et al. (49) multiplex PCR method for differentiating *L. monocytogenes* serogroups. All four strains contained a complete *prs* sequence (the loci from the scheme that is common to all *Listeria* species); eight mismatches were identified in all but one of the strains, FSL L7-0020, which had nine. Additionally, no primer mismatches were detected with all four *L. swaminathanii prs* sequences when conducting an *in silico* PCR analysis, suggesting these strains would be identified as *Listeria* sp. by the multiplex PCR method. Additionally, there were no hits for the four *L. monocytogenes* specific loci from the scheme, so the *L. swaminathanii* strains were not given a serogroup designation or incorrectly identified as *L. monocytogenes*.

### Virulence and resistance genes

To support the phenotypic absence of pathogenic characteristics, the *L. swaminathanii* genomes were screened for genes involved in virulence. The key genes required for pathogenicity (i.e., the genes found in the *Listeria* Pathogenicity Island-1 [LIPI-1; *prfA*, *plcA*, *hly*, *mpl*, *actA*, and *plcB*] and the internalin genes [*inlA* and *inlB*]) (50–52) were absent for all four genomes (Table S6). Notably, the absence of *plcA* and *hly*, the genes that confer PI-PLC activity and β-hemolysis, respectively, supports the lack of virulence characteristics described above. The genes associated with LIPI-3 (*llsABDGPX*Y) were found in UTK C1-0024; however, the absence of LIPI-1, *inlA*, and *inlB* supports that *L. swaminathanii* is nonpathogenic. The D-aminopeptidase coding sequences were absent from the *L. swaminathanii* genomes and present in the *L. innocua* ATCC 33090^T^ DIM-positive reference genome, which supports the absence of D-arylamidase activity (i.e., DIM-negative *Listeria* API result).

The four genomes representing *L. swaminathanii* were also screened for loci associated with antimicrobial or disinfectant resistance. All yielded strict hits for *L. monocytogenes mprF*, *fosX*, *vanT* in the *vanG* cluster, and *vanY* in the *vanB* cluster. *mprF* and *fosX* are both part of the *L. monocytogenes* core genome and confer resistance to cationic peptides (including defensins) and fosfomycin, respectively (53). The *vanG* and *vanB* clusters are inducible clusters that confer resistance to vancomycin (54, 55). UTK S2-0008^T^ and FSL L7-0020 also yielded a strict hit for *norB*, and FSL L7-0020 and UTK C1-0015 both yielded a strict hit for *lin. norB* and *lin* are also part of the *L. monocytogenes* core genome and confer resistance to quinolones (including nalidixic acid) and lincosamides (including clindamycin), respectively (53). No detergent resistance genes (*qac*, *bcrABC*, or *ermEC*; which have been reported as conferring reduced quaternary ammonium sensitivity [56]) were detected.

### Conclusions

Reassignment of the type strain *for Listeria swaminathanii* is necessary because the originally designated type strain, FSL L7-0020, could not achieve valid publication status due to restrictions associated with its isolation from a U.S. National Park. In addition, its atypical catalase-negative phenotype suggests that it may not represent the species as accurately as other strains. The proposed type strain, UTK S2-0008^T^, meets all accessibility requirements, and ORGIs (ANI and *is*DDH) confirm species-level delineation. UTK S2-0008^T^ exhibits phenotypic characteristics consistent with the *Listeria sensu stricto* clade, including growth at 4°C and motility at 25°C. Genotypic and phenotypic characterization also indicate that it is nonpathogenic. Valid publication of *L. swaminathanii* is important for food safety monitoring and diagnostic method validation. Specifically, inclusion of this species in reference databases and inclusivity studies will be critical to ensure accurate identification and detection across regulatory and industry applications.

### Description of *Listeria swaminathanii* sp. nov.

*L. swaminathanii* (swa.mi.na.tha´ni.i N.L. gen. n. *swaminathanii*, named in honor of Balasubramanian Swaminathan for his contributions to the epidemiology of human listeriosis and laboratory diagnostic methodologies).

*L. swaminathanii* exhibits the growth characteristics typical of nonpathogenic *Listeria sensu stricto* spp. Gram-positive short rods. Oxidase-negative. Catalase-positive. Methyl red-positive. Voges-Proskauer-positive. Facultative anaerobe. Motile at 25°C, non-motile at 37°C. Growth occurs between 4°C and 41°C in brain heart infusion (BHI) broth, with optimal growth achieved between 30°C and 37°C. Esculin-positive. Presumed to be nonpathogenic based on the absence of hemolysis on SBA, lack of PI-PLC activity on ALOA, and the absence of six virulence genes (*prfA*, *plcA*, *hly*, *mpl*, *actA*, and *plcB*) located on LIPI-1. Colonies on MOX are small, round, black, with a sunken center. Colonies on ALOA were similar in size and shape to those on MOX and were blue-green in color. Does not reduce nitrate or nitrite. Does not ferment glucose-1-phosphate, erythritol, D-arabinose, L-arabinose, D-ribose, D-xylose, D-adonitol, methyl-β-D-xylopyranoside, D-galactose, L-sorobose, L-rhamnose, dulcitol, inositol, D-mannitol, D-sorbitol, D-melibiose, sucrose, inulin, D-melezitose, D-raffinose, starch, glycogen, D-turanose, D-lyxose, D-tagatose, D-fucose, L-arabitol, potassium gluconate, potassium 2-ketogluconate, and potassium 5-ketogluconate. Positive for the fermentation of D-glucose, D-fructose, D-mannose, methyl-α-D-glucopyranoside, methyl-α-D-mannopyranoside, N-acetylglucosamine, amygdalin, arbutin, salicin, D-cellobiose, D-maltose, D-lactose, xylitol, and gentibiose. Variable fermentation of glycerol (UTK C1-0015, UTK C1-0024 does not ferment), D-lactose (UTK C1-0015 does not ferment), and D-trehalose (FSL L7-0020, UTK C1-0024 does not ferment).

The type strain, UTK S2-0008^T^, was isolated in September 2022 from soil collected in the Nantahala National Forest. The genomic DNA G + C content is 38.5 mol%, and the complete genome has a total length of 2.8 Mb. This strain is deposited in the Culture Collection University of Gothenburg (CCUG 77280^T^) and the Belgian Coordinated Collection of Microbiology (LMG 33255^T^).

## MATERIALS AND METHODS

### Strain collection and isolation

A study conducted by Wang et al. (57) evaluating the diversity of *Listeria* in the Nantahala National Forest, North Carolina, USA, yielded 12 *Listeria*-like isolates from soil samples. *Listeria* colonies were isolated using a modified U.S. Food and Drug Administration’s *Bacteriological Analytical Manual* (FDA BAM) Chapter 10 method (46), as previously described by Wang et al. (57). Frozen stock cultures were prepared by transferring 1 mL of the BHI broth culture to BHI broth containing 15% glycerol (vol/vol), followed by storage at −80°C.

### Whole-genome sequencing

The 12 *Listeria-*like isolates were short-read sequenced and analyzed by Wang et al. (57) and determined to be identical. Briefly, high-quality single-nucleotide polymorphism (hqSNP) distances were evaluated using the CFSAN SNP Pipeline (v2.2.1) (58) and found to be 0 hqSNPs apart. The draft genomes all met the quality standards proposed by Chun et al. (41) for taxonomic classification of prokaryotes, which include (i) N50 >50,000 bp (N50 = 1.4 Mb for all genomes), (ii) total number of contigs <300 (maximum of 11 contigs), (iii) average coverage >30× (average coverage is >150×; Table S1). *L. swaminathanii* UTK S2-0008^T^ was selected as the type strain and proceeded to long-read sequencing and further genomic and phenotypic analyses.

A complete genome for UTK S2-0008^T^ was generated through long-read sequencing and hybrid assembly. Genomic DNA was extracted using the Qiagen DNA Easy Mini Kit (Qiagen GmbH) according to the manufacturer’s instructions. The Rapid Barcoding Kit (SQK-RBK114.24, V14; Oxford Nanopore Technologies, Oxford, UK) was used for library preparation per the manufacturer’s instructions, but substituting Magbio Genomics Inc HighPrep PCR SPRI beads for AMPure XP SPRI beads. Sequencing was performed on an Oxford Nanopore MinIon instrument with a Flongle adapter (ADP-FLG001) and Flongle flowcell (FLO-FLG114, R10 version). MinKNOW software (v5.8.7) was used for basecalling (fast basecalling model). Unicycler (v0.4.8) was used to generate a hybrid assembly using both the long reads and previously described Illumina reads (57). The raw reads were submitted to NCBI SRA (SRR29203347), and the assembly was submitted to NCBI (GCA_031843265.2) and annotated using the NCBI Prokaryotic Genome Annotation Pipeline (PGAP; v6.7).

### ORGIs calculations

ANIb analysis included the complete genome of *L. swaminathanii* UTK S2-0008^T^ and two previously characterized *L. swaminathanii* strains (UTK C1-0015 and UTK C1-0024), the draft genome of the *L. swaminathanii* strain FSL L7-0020, and a set of 35 *Listeria* reference genomes (Fig. 1). The reference set included one genome for each of the four *L. monocytogenes* lineages, along with the type strains for all validly published species and subspecies characterized as of 6 January 2026. ANIb was completed using pyani (v0.2.7) (59). The ANI-based dendrogram of the pyani output was created with the dendextend R package (60). To further support the ANIb identification, *is*DDH was conducted between *L. marthii* FSL S4-120^T^ and all *L. swaminathanii* genomes. The isDDH values were determined using the Genome-to-Genome Distance Calculator 3.0, formula 2 (identities/high-scoring segment pair) available from Leibniz Institute DSMZ (61).

### Phenotypic characterization

Phenotypic characterization was performed on *L. swaminathanii* UTK S2-0008^T^ to assess the following characteristics: growth across the growth range expected for *Listeria* (4°C–41°C) (1), growth under anaerobic conditions, colony phenotypes on selective and differential media, motility, Gram stain, hemolysis, oxidase, catalase, nitrate reduction, Methyl Red-Voges Proskauer, and the biochemical tests included in the API 50CH kits (bioMérieux, Marcy-l’Étoile, France). Identification by four rapid *Listeria* species identification methods was also assessed: API *Listeria* (bioMérieux), VITEK 2 (bioMérieux), VITEK MS (bioMérieux), and MALDI Biotyper (Bruker, Billerica, MA, USA). The carbohydrate utilization tests historically used to differentiate *Listeria* species (rhamnose, xylose, and mannitol) were assessed with both API *Listeria* and API 50 CH (46, 47). The phenotypic analyses were conducted using pure cultures grown aerobically on BHI agar at 30°C for 24 h. Three biological replicates were performed for the growth temperature experiments, growth under anaerobic conditions, and the catalase test; two biological replicates were performed for all other characterization and rapid *Listeria* species identification methods. With the exception of the oxidase, Methyl Red, Voges-Proskauer, and MALDI Biotyper analyses, all phenotypic characterization tests were conducted as per the standardized *Listeria* identification procedures described in the FDA BAM Chapter 10 and ISO EN 11290-1:2017 (both documents describe the same tests), and as previously described by Carlin et al. (22, 23, 37) and Hudson et al. (38).

*L. monocytogenes* 10403S was included as a positive control for the growth temperature experiments, motility, catalase test, hemolysis test, and VP test and as a negative control for nitrate reduction. *L. monocytogenes* ATCC 43257 was included as a positive control for the catalase test, hemolysis test, and VP test; as a negative control for PI-PLC activity, oxidase test, and MR test; and as a control for the API 50 CH. *L. innocua* ATCC 33090^T^ was included as a negative control for PI-PLC activity. *L. booriae* FSL A5-0281^T^ was included as a positive control for nitrate reduction and a negative control for motility, catalase test, hemolysis test, and VP test. *L. marthii* FSL S4-120^T^, *L. cossartiae* subsp. *cossartiae* FSL L7-1447^T^, *L. cossartiae* subsp. *cayugensis* FSL L7-0993^T^, and *L. swaminathanii* FSL L7-0020 were included as controls for the API 50 CH. *Pseudomonas aeruginosa* ATCC 9027 was included as a positive control for the oxidase and MR tests.

Growth temperature experiments were conducted at 4°C, 7°C, 22°C, 30°C, 37°C, and 41°C. Growth under anaerobic conditions was conducted at 30°C. Motility was assessed by (i) inoculating motility test medium (BD) and (ii) microscopic observation at two temperatures, 25°C and 37°C. Colony morphology, esculin hydrolysis, and PI-PLC enzyme activity were assessed by performing a three-phase streak of overnight BHI broth cultures onto MOX and ALOA (bioMérieux).

Oxidase activity was assessed using oxidase reagent droppers (BD) according to the manufacturer’s instructions (62). The MR-VP test was performed as described by Powers and Latt (63). Briefly, growth from BHI agar was streaked onto modified MR-VP agar (one plate for each test), followed by incubation at 35°C for 24 h. Methyl red reagent (Thermo Fisher Scientific) was added to the growth on one plate for the MR test, and VP1 and VP2 reagents (bioMérieux) were added to the other for the VP test. *L. swaminathanii* UTK S2-0008^T^ was further characterized using API 50 CH with incubation at 35°C under aerobic conditions for up to 4 days. The VITEK 2 (v 9.02) (64), VITEK MS (v 3.2.0) (65), and MALDI Biotyper (v 4.1.100) (66) rapid automated identification methods were performed according to the manufacturer’s instructions on *L. swaminathanii* UTK S2-008^T^ and FSL L7-0070.

### Additional genomic characterization

The *L. swaminathanii* UTK S2-0008^T^, UTK C1-0015, and UTK C1-024 closed genomes and FSL L7-0020 draft genome were further analyzed for the presence of *L. monocytogenes* serogroup-specific genes, virulence genes, antimicrobial resistance (AMR) genes, and sanitizer resistance genes.

The four *L. swaminathanii* genomes were analyzed using the PCR serogroup scheme from the Institut Pasteur database, BIGSdb-*Lm* (49, 67); this database consists of alleles from the five loci (*lmo0737*, *lmo1118*, ORF2819, ORF2110, and *prs*) targeted by the Doumith et al. (49) multiplex PCR method for differentiating *L. monocytogenes* serogroups. The *prs* sequence (the target common to all *Listeria* species) for each strain was compared to the BIGSdb-*Lm* reference using BLASTn (68). An *in silico* PCR analysis was conducted using the NCBI Primer BLAST tool (69) and the primers described by Doumith et al. (49).

Pathogenic potential was assessed by querying the *L. swaminathanii* genomes against the virulence scheme from the Institut Pasteur database, BIGSdb-*Lm* (67), which contains a set of 92 loci. The D-arylamidase activity was assessed by searching for D-aminopeptidase coding sequences (WP_003759899.1) as previously described by Carlin et al. (22, 23). AMR was assessed using the Resistance Gene Identifier (RGI 6.0.3) software, which uses the Comprehensive Antimicrobial Resistance Database (CARD 3.2.9) to predict resistomes. RGI was accessed via the web portal (https://card.mcmaster.ca/analyze/rgi) with the criteria for perfect and strict hits only (70). Using the Institut Pasteur disinfectant resistance gene database (*qac*, *bcrABC*, and *ermEC*) (67), the draft genomes were queried using blastn and megablast (68).

## Data Availability

The GenBank/EMBL/DDBJ accession numbers for the 16S rRNA sequence and complete genome of UTK S2-0008^T^ are PP860874 and GCA_031843265.2, respectively. The SRA accession numbers for the raw sequencing data for UTK S2-0008^T^ are SRR24374380 and SRR29203347. UTK S2-0008^T^ is deposited in the Culture Collection University of Gothenburg (CCUG 77280^T^) and the Belgian Coordinated Collection of Microbiology (LMG 33255^T^).

## SUPPLEMENTAL MATERIAL

Supplemental tables spectrum.04139-25-s0001.xlsx Tables S1 to S6.

ASM does not own the copyrights to Supplemental Material that may be linked to, or accessed through, an article. The authors have granted ASM a non-exclusive, world-wide license to publish the Supplemental Material files. Please contact the corresponding author directly for reuse.

## References

[1] McLauchlin J, Rees CED. 2015. *Listeria*, p 1–29. *In* Trujillo ME, Dedysh S, DeVos P, Hedlund B, Kämpfer P, Rainey FA, Whitman WB (ed), Bergey’s manual of systematics of archaea and bacteria. John Wiley & Sons, Inc.

[2] Ludwig W, Schleifer K-H, Whitman WB. 2015. Listeriaceae” fam, p 1. *In* Trujillo ME, Dedysh S, DeVos P, Hedlund B, Kämpfer P, Rainey FA, Whitman WB (ed), Bergey’s manual of systematics of archaea and bacteria. John Wiley & Sons, Inc.

[3] Sneath PHA. 2015. *Brochothrix*, p 1–9. *In* Trujillo ME, Dedysh S, DeVos P, Hedlund B, Kämpfer P, Rainey FA, Whitman WB (ed), Bergey’s manual of systematics of archaea and bacteria. John Wiley & Sons, Inc.

[4] Ryser ET, Arimi SM, Donnelly CW. 1997. Effects of pH on distribution of *Listeria* ribotypes in corn, hay, and grass silage. Appl Environ Microbiol 63:3695–3697. doi:10.1128/aem.63.9.3695-3697.1997 9293020 PMC168675

[5] Orsi RH, Liao J, Carlin CR, Wiedmann M. 2024. Taxonomy, ecology, and relevance to food safety of the genus *Listeria* with a particular consideration of new *Listeria* species described between 2010 and 2022. mBio 15:e0093823. doi:10.1128/mbio.00938-23 38126771 PMC10865800

[6] Liao J, Guo X, Weller DL, Pollak S, Buckley DH, Wiedmann M, Cordero OX. 2021. Nationwide genomic atlas of soil-dwelling *Listeria* reveals effects of selection and population ecology on pangenome evolution. Nat Microbiol 6:1021–1030. doi:10.1038/s41564-021-00935-7 34267358

[7] Malley TJV, Butts J, Wiedmann M. 2015. Seek and destroy process: *Listeria monocytogenes* process controls in the ready-to-eat meat and poultry industry. J Food Prot 78:436–445. doi:10.4315/0362-028X.JFP-13-507 25710164

[8] Alali WQ, Schaffner DW. 2013. Relationship between *Listeria monocytogenes* and *Listeria* spp. in seafood processing plants. J Food Prot 76:1279–1282. doi:10.4315/0362-028X.JFP-13-030 23834807

[9] Ryser ET, Marth EH. 2007. Listeria, listeriosis, and food safety. CRC press.

[10] Murray EGD, Webb RA, Swann MBR. 1926. A disease of rabbits characterised by a large mononuclear leucocytosis, caused by a hitherto undescribed bacillus *Bacterium monocytogenes* (n.sp.). J Pathol 29:407–439. doi:10.1002/path.1700290409

[11] Parte AC, Sardà Carbasse J, Meier-Kolthoff JP, Reimer LC, Göker M. 2020. List of prokaryotic names with standing in nomenclature (LPSN) moves to the DSMZ. Int J Syst Evol Microbiol 70:5607–5612. doi:10.1099/ijsem.0.004332 32701423 PMC7723251

[12] Havelaar AH, Kirk MD, Torgerson PR, Gibb HJ, Hald T, Lake RJ, Praet N, Bellinger DC, de Silva NR, Gargouri N, et al.. 2015. World health organization global estimates and regional comparisons of the burden of foodborne disease in 2010. PLoS Med 12:e1001923. doi:10.1371/journal.pmed.1001923 26633896 PMC4668832

[13] Seeliger HP, Rocourt J, Schrettenbrunner A, Grimont P, Jones D. 1984. Notes: *Listeria ivanovii* sp. nov. Int J Syst Bacteriol 34:336–337. doi:10.1099/00207713-34-3-336

[14] Boerlin P, Rocourt J, Grimont F, Grimont PA, Jacquet C, Piffaretti J-C. 1992. *Listeria ivanovii* subsp. *londoniensis* subsp. nov. Int J Syst Bacteriol 42:69–73. doi:10.1099/00207713-42-1-69 1736964

[15] Guillet C, Join-Lambert O, Le Monnier A, Leclercq A, Mechaï F, Mamzer-Bruneel M-F, Bielecka MK, Scortti M, Disson O, Berche P, et al.. 2010. Human listeriosis caused by *Listeria ivanovii*. Emerging Infectious Disease journal 16:136. doi:10.3201/eid1601.091155 PMC2874378 20031061

[16] Gouin E, Mengaud J, Cossart P. 1994. The virulence gene cluster of *Listeria monocytogenes* is also present in *Listeria ivanovii*, an animal pathogen, and *Listeria seeligeri*, a nonpathogenic species. Infect Immun 62:3550–3553. doi:10.1128/iai.62.8.3550-3553.1994 8039927 PMC302991

[17] Orsi RH, Wiedmann M. 2016. Characteristics and distribution of *Listeria* spp., including *Listeria* species newly described since 2009. Appl Microbiol Biotechnol 100:5273–5287. doi:10.1007/s00253-016-7552-2 27129530 PMC4875933

[18] Chiara M, Caruso M, D’Erchia AM, Manzari C, Fraccalvieri R, Goffredo E, Latorre L, Miccolupo A, Padalino I, Santagada G, et al.. 2015. Comparative genomics of *Listeria sensu lato*: genus-wide differences in evolutionary dynamics and the progressive gain of complex, potentially pathogenicity-related traits through lateral gene transfer. Genome Biol Evol 7:2154–2172. doi:10.1093/gbe/evv131 26185097 PMC4558849

[19] Seeliger H. 1981. Nonpathogenic listeriae: *L. innocua* sp. n. (Seeliger et schoofs, 1977) (authors transl). Zentralbl Bakteriol Mikrobiol Hyg A 249:487–493. doi:10.1016/S0174-3031(81)80108-4 6171948

[20] Rocourt J, Grimont PAD. 1983. Notes: *Listeria welshimeri* sp. nov. and *Listeria seeligeri* sp. nov. Int J Syst Bacteriol 33:866–869. doi:10.1099/00207713-33-4-866

[21] Graves LM, Helsel LO, Steigerwalt AG, Morey RE, Daneshvar MI, Roof SE, Orsi RH, Fortes ED, Milillo SR, den Bakker HC, et al.. 2010. *Listeria marthii* sp. nov., isolated from the natural environment, finger lakes national forest. Int J Syst Evol Microbiol 60:1280–1288. doi:10.1099/ijs.0.014118-0 19667380

[22] Carlin CR, Liao J, Weller DL, Guo X, Orsi R, Wiedmann M. 2021. Corrigendum: *Listeria cossartiae* sp. nov., *Listeria farberi* sp. nov., *Listeria immobilis* sp. nov., *Listeria portnoyi* sp. nov. and *Listeria rustica* sp. nov., isolated from agricultural water and natural environments. Int J Syst Evol Microbiol 71. doi:10.1099/ijsem.0.004885 PMC8289207 33999788

[23] Carlin CR, Liao J, Weller D, Guo X, Orsi R, Wiedmann M. 2021. Listeria cossartiae sp. nov., Listeria immobilis sp. nov., Listeria portnoyi sp. nov. and Listeria rustica sp. nov., isolated from agricultural water and natural environments. Int J Syst Evol Microbiol 71:004795. doi:10.1099/ijsem.0.004795 33999788 PMC8289207

[24] den Bakker HC, Warchocki S, Wright EM, Allred AF, Ahlstrom C, Manuel CS, Stasiewicz MJ, Burrell A, Roof S, Strawn LK, et al.. 2014. *Listeria floridensis* sp. nov., *Listeria aquatica* sp. nov., *Listeria cornellensis* sp. nov., *Listeria riparia* sp. nov. and *Listeria grandensis* sp. nov., from agricultural and natural environments. Int J Syst Evol Microbiol 64:1882–1889. doi:10.1099/ijs.0.052720-0 24599893

[25] Bertsch D, Rau J, Eugster MR, Haug MC, Lawson PA, Lacroix C, Meile L. 2013. *Listeria fleischmannii* sp. nov., isolated from cheese. Int J Syst Evol Microbiol 63:526–532. doi:10.1099/ijs.0.036947-0 22523164

[26] den Bakker HC, Manuel CS, Fortes ED, Wiedmann M, Nightingale KK. 2013. Genome sequencing identifies *Listeria fleischmannii* subsp. *coloradonensis* subsp. nov., isolated from a ranch. Int J Syst Evol Microbiol 63:3257–3268. doi:10.1099/ijs.0.048587-0 23524352

[27] Doijad SP, Poharkar KV, Kale SB, Kerkar S, Kalorey DR, Kurkure NV, Rawool DB, Malik SVS, Ahmad RY, Hudel M, et al.. 2018. *Listeria goaensis* sp. nov. Int J Syst Evol Microbiol 68:3285–3291. doi:10.1099/ijsem.0.002980 30156532

[28] Leclercq A, Moura A, Vales G, Tessaud-Rita N, Aguilhon C, Lecuit M. 2019. *Listeria thailandensis* sp. nov. Int J Syst Evol Microbiol 69:74–81. doi:10.1099/ijsem.0.003097 30457511

[29] Quereda JJ, Leclercq A, Moura A, Vales G, Gómez-Martín Á, García-Muñoz Á, Thouvenot P, Tessaud-Rita N, Bracq-Dieye H, Lecuit M. 2020. *Listeria valentina* sp. nov., isolated from a water trough and the faeces of healthy sheep. Int J Syst Evol Microbiol 70:5868–5879. doi:10.1099/ijsem.0.004494 33016862

[30] Núñez-Montero K, Leclercq A, Moura A, Vales G, Peraza J, Pizarro-Cerdá J, Lecuit M. 2018. *Listeria costaricensis* sp. nov. Int J Syst Evol Microbiol 68:844–850. doi:10.1099/ijsem.0.002596 29458479

[31] Raufu IA, Moura A, Vales G, Ahmed OA, Aremu A, Thouvenot P, Tessaud-Rita N, Bracq-Dieye H, Krishnamurthy R, Leclercq A, et al.. 2022. *Listeria ilorinensis* sp. nov., isolated from cow milk cheese in Nigeria. Int J Syst Evol Microbiol 72:005437. doi:10.1099/ijsem.0.005437 35731854

[32] Rocourt J, Boerlin P, Grimont F, Jacquet C, Piffaretti J-C. 1992. Assignment of *Listeria grayi* and *Listeria murrayi* to a single species, *Listeria grayi*, with a revised description of *Listeria grayi*. Int J Syst Bacteriol 42:171–174. doi:10.1099/00207713-42-1-171 1736964

[33] Leclercq A, Clermont D, Bizet C, Grimont PAD, Le Flèche-Matéos A, Roche SM, Buchrieser C, Cadet-Daniel V, Le Monnier A, Lecuit M, et al.. 2010. *Listeria rocourtiae* sp. nov. Int J Syst Evol Microbiol 60:2210–2214. doi:10.1099/ijs.0.017376-0 19915117

[34] Lang Halter E, Neuhaus K, Scherer S. 2013. *Listeria weihenstephanensis* sp. nov., isolated from the water plant *Lemna trisulca* taken from a freshwater pond. Int J Syst Evol Microbiol 63:641–647. doi:10.1099/ijs.0.036830-0 22544790

[35] Weller D, Andrus A, Wiedmann M, den Bakker HC. 2015. *Listeria booriae* sp. nov. and *Listeria newyorkensis* sp. nov., from food processing environments in the USA. Int J Syst Evol Microbiol 65:286–292. doi:10.1099/ijs.0.070839-0 25342111

[36] Brown P, Moura A, Valès G, Tessaud-Rita N, Niedermeyer J, Parsons C, Leclercq A, Harris A, Emanuel RE, Kathariou S, et al.. 2025. *Listeria tempestatis* sp. nov. and *Listeria rocourtiae* subsp. *hofi* subsp. nov. Int J Syst Evol Microbiol 75. doi:10.1099/ijsem.0.006774 PMC12378429 40358009

[37] Carlin CR, Liao J, Hudson LK, Peters TL, Denes TG, Orsi RH, Guo X, Wiedmann M. 2022. Soil collected in the Great Smoky Mountains National Park yielded a novel *Listeria sensu stricto* species, *L. swaminathanii*. Microbiol Spectr 10:e0044222. doi:10.1128/spectrum.00442-22 35658601 PMC9241783

[38] Hudson LK, Chaggar HK, Schamp CN, Claxton ML, Bryan DW, Peters TL, Song Y, Carlin CR, den Bakker HC, Denes TG. 2022. Phenotypic characterization and analysis of complete genomes of two distinct strains of the proposed species “*L. swaminathanii*”. Sci Rep 12:9137. doi:10.1038/s41598-022-13119-y 35650389 PMC9159981

[39] . Available from: https://www.the-icsp.org/index.php/minutes-reports-2020-2023. Retrieved 1818 JunJune 2026. Accessed , 1818 JunJune 2026

[40] Oren A, Arahal DR, Göker M, Moore ERB, Rossello-Mora R, Sutcliffe IC. 2023. International code of nomenclature of prokaryotes. prokaryotic code (2022 Revision). Int J Syst Evol Microbiol 73:005585. doi:10.1099/ijsem.0.005585 37219928

[41] Chun J, Oren A, Ventosa A, Christensen H, Arahal DR, da Costa MS, Rooney AP, Yi H, Xu X-W, De Meyer S, et al.. 2018. Proposed minimal standards for the use of genome data for the taxonomy of prokaryotes. Int J Syst Evol Microbiol 68:461–466. doi:10.1099/ijsem.0.002516 29292687

[42] Tindall BJ, Rosselló-Móra R, Busse H-J, Ludwig W, Kämpfer P. 2010. Notes on the characterization of prokaryote strains for taxonomic purposes. Int J Syst Evol Microbiol 60:249–266. doi:10.1099/ijs.0.016949-0 19700448

[43] Riesco R, Trujillo ME. 2024. Update on the proposed minimal standards for the use of genome data for the taxonomy of prokaryotes. Int J Syst Evol Microbiol 74:006300. doi:10.1099/ijsem.0.006300 38512750 PMC10963913

[44] Goris J, Konstantinidis KT, Klappenbach JA, Coenye T, Vandamme P, Tiedje JM. 2007. DNA-DNA hybridization values and their relationship to whole-genome sequence similarities. Int J Syst Evol Microbiol 57:81–91. doi:10.1099/ijs.0.64483-0 17220447

[45] Parks DH, Chaumeil P-A, Mussig AJ, Rinke C, Chuvochina M, Hugenholtz P. 2026. GTDB release 10: a complete and systematic taxonomy for 715 230 bacterial and 17 245 archaeal genomes. Nucleic Acids Res 54:D743–D754. doi:10.1093/nar/gkaf1040 41123020 PMC12807784

[46] Hitchins AD, Jinneman K, Chen Y. 2017. Chapter 10, Detection of Listeria monocytogenes in foods and environmental samples, and enumeration of Listeria monocytogenes in foods. *In* U.S. food and drug administration (ed), bacteriological analytical manual (BAM)

[47] International Organization for Standardization. 2017. Microbiology of food and animal feeding stuffs—horizontal method for the detection and enumeration of Listeria monocytogenes and Listeria spp, p 11290–11291. ISO, Geneva.

[48] Zhao H, Chen J, Fang C, Xia Y, Cheng C, Jiang L, Fang W. 2011. Deciphering the biodiversity of *Listeria monocytogenes* lineage III strains by polyphasic approaches. J Microbiol 49:759–767. doi:10.1007/s12275-011-1006-4 22068492

[49] Doumith M, Buchrieser C, Glaser P, Jacquet C, Martin P. 2004. Differentiation of the major *Listeria monocytogenes* serovars by multiplex PCR. J Clin Microbiol 42:3819–3822. doi:10.1128/JCM.42.8.3819-3822.2004 15297538 PMC497638

[50] Orsi RH, Borowsky ML, Lauer P, Young SK, Nusbaum C, Galagan JE, Birren BW, Ivy RA, Sun Q, Graves LM, et al.. 2008. Short-term genome evolution of *Listeria monocytogenes* in a non-controlled environment. BMC Genomics 9:539. doi:10.1186/1471-2164-9-539 19014550 PMC2642827

[51] Jacquet C, Doumith M, Gordon JI, Martin PMV, Cossart P, Lecuit M. 2004. A molecular marker for evaluating the pathogenic potential of foodborne *Listeria monocytogenes*. J Infect Dis 189:2094–2100. doi:10.1086/420853 15143478

[52] Dussurget O, Pizarro-Cerda J, Cossart P. 2004. Molecular determinants of *Listeria monocytogenes* virulence. Annu Rev Microbiol 58:587–610. doi:10.1146/annurev.micro.57.030502.090934 15487949

[53] Moura A, Leclercq A, Vales G, Tessaud-Rita N, Bracq-Dieye H, Thouvenot P, Madec Y, Charlier C, Lecuit M. 2024. Phenotypic and genotypic antimicrobial resistance of *Listeria monocytogenes*: an observational study in France. Lancet Reg Health Eur 37:100800. doi:10.1016/j.lanepe.2023.100800 38362545 PMC10866989

[54] The comprehensive antibiotic resistance database (CARD). 2022. Glycopeptide Resistance Gene Cluster VanG. Available from: https://card.mcmaster.ca/ontology/36396

[55] The comprehensive antibiotic resistance database (CARD). 2022. Glycopeptide Resistance Gene Cluster VanB. Available from: https://card.mcmaster.ca/ontology/36377

[56] Møretrø T, Schirmer BCT, Heir E, Fagerlund A, Hjemli P, Langsrud S. 2017. Tolerance to quaternary ammonium compound disinfectants may enhance growth of *Listeria monocytogenes* in the food industry. Int J Food Microbiol 241:215–224. doi:10.1016/j.ijfoodmicro.2016.10.025 27810443

[57] Wang J, Schamp CN, Hudson LK, Chaggar HK, Bryan DW, Garman KN, Radosevich M, Denes TG. 2025. Whole-genome sequencing and metagenomics reveal diversity and prevalence of *Listeria* spp. from soil in the Nantahala National Forest. Microbiol Spectr 13:e01712–24. doi:10.1128/spectrum.01712-24 39651889 PMC11705966

[58] Davis S, Pettengill JB, Luo Y, Payne J, Shpuntoff A, Rand H, Strain E. 2015. CFSAN SNP pipeline: an automated method for constructing SNP matrices from next-generation sequence data. PeerJ Comput Sci 1:e20. doi:10.7717/peerj-cs.20

[59] Pritchard L, Glover RH, Humphris S, Elphinstone JG, Toth IK. 2016. Genomics and taxonomy in diagnostics for food security: soft-rotting enterobacterial plant pathogens. Anal Methods 8:12–24. doi:10.1039/C5AY02550H

[60] Galili T. 2015. Dendextend: an R package for visualizing, adjusting and comparing trees of hierarchical clustering. Bioinformatics 31:3718–3720. doi:10.1093/bioinformatics/btv428 26209431 PMC4817050

[61] Meier-Kolthoff JP, Auch AF, Klenk H-P, Göker M. 2013. Genome sequence-based species delimitation with confidence intervals and improved distance functions. BMC Bioinformatics 14:1–14. doi:10.1186/1471-2105-14-60 23432962 PMC3665452

[62] Becton Dickinson and Company. 2022. BD BBL oxidase reagent droppers, REF 261181 (Instructions for use [IFU]). Sparks, Maryland, USA

[63] Powers EM, Latt TG. 1977. Simplified 48-hour IMVic test: an agar plate method. Appl Environ Microbiol 34:274–279. doi:10.1128/aem.34.3.274-279.1977 334074 PMC242642

[64] bioMeriéux. 2015. User manual - 514740-2EN1 - VITEK 2 systems product information

[65] bioMeriéux. 2020. User manual supplements - 161150-923 - A1 - en - VITEK MS industry use - V3.2 knowledge base

[66] Bastin B, Bird P, Crowley E, Benzinger MJ, Agin J, Goins D, Sohier D, Timke M, Awad M, Kostrzewa M. 2018. Confirmation and identification of *Listeria monocytogenes, Listeria* spp. and other gram-positive organisms by the bruker MALDI biotyper method: collaborative study, first action 2017.10. J AOAC Int 101:1610–1622. doi:10.5740/jaoacint.18-0013 29703273

[67] Moura A, Criscuolo A, Pouseele H, Maury MM, Leclercq A, Tarr C, Björkman JT, Dallman T, Reimer A, Enouf V, et al.. 2016. Whole genome-based population biology and epidemiological surveillance of *Listeria monocytogenes*. Nat Microbiol 2:16185. doi:10.1038/nmicrobiol.2016.185 27723724 PMC8903085

[68] Zhang Z, Schwartz S, Wagner L, Miller W. 2000. A greedy algorithm for aligning DNA sequences. J Comput Biol 7:203–214. doi:10.1089/10665270050081478 10890397

[69] Ye J, Coulouris G, Zaretskaya I, Cutcutache I, Rozen S, Madden TL. 2012. Primer-BLAST: a tool to design target-specific primers for polymerase chain reaction. BMC Bioinformatics 13:1–11. doi:10.1186/1471-2105-13-134 22708584 PMC3412702

[70] Alcock BP, Huynh W, Chalil R, Smith KW, Raphenya AR, Wlodarski MA, Edalatmand A, Petkau A, Syed SA, Tsang KK, et al.. 2023. CARD 2023: expanded curation, support for machine learning, and resistome prediction at the comprehensive antibiotic resistance database. Nucleic Acids Res 51:D690–D699. doi:10.1093/nar/gkac920 36263822 PMC9825576

